# *iq*: an R package to estimate relative protein abundances from ion quantification in DIA-MS-based proteomics

**DOI:** 10.1093/bioinformatics/btz961

**Published:** 2020-01-07

**Authors:** Thang V Pham, Alex A Henneman, Connie R Jimenez

**Affiliations:** OncoProteomics Laboratory, Department of Medical Oncology, Cancer Center Amsterdam, Amsterdam UMC, Vrije Universiteit Amsterdam, Amsterdam 1081 HV, The Netherlands

## Abstract

**Summary:**

We present an R package called *iq* to enable accurate protein quantification for label-free data-independent acquisition (DIA) mass spectrometry-based proteomics, a recently developed global approach with superior quantitative consistency. We implement the popular maximal peptide ratio extraction module of the MaxLFQ algorithm, so far only applicable to data-dependent acquisition mode using the software suite MaxQuant. Moreover, our implementation shows, for each protein separately, the validity of quantification over all samples. Hence, *iq* exports a state-of-the-art protein quantification algorithm to the emerging DIA mode in an open-source implementation.

**Availability and implementation:**

The open-source R package is available on CRAN, https://github.com/tvpham/iq/releases and oncoproteomics.nl/iq.

**Supplementary information:**

Supplementary data are available at *Bioinformatics* online.

## 1 Introduction

The data-independent acquisition (DIA) approach in mass spectrometry (MS)-based proteomics has emerged as a promising alternative to data-dependent acquisition (DDA) because of its ability to provide a more complete data matrix by combining unbiased, broad range precursor ion fragmentation and targeted data extraction (Gillet *et al.*, 2012). Since in both approaches, protein abundances are derived from multiple partial intensities, peptide precursor and peptide fragment intensities in DDA and DIA, respectively, it is desirable to extend advanced DDA quantification methods and insights to DIA data, which possesses the largest degree of multiplicity. 

Existing methods to estimate protein abundances for DIA data include the so-called topN approach where the top *N* most intense ions are aggregated, either by summation or averaging, as they tend to be more robust than less intense ions. The MeanInt approach averages all extracted ion intensities (Ahrné *et al.*, 2013). Another method is the median polish algorithm (Tukey, 1977), available as part of the standard R distribution and employed by the package MSstats (Choi *et al.*, 2014). For DDA data, the MaxLFQ algorithm elegantly combines multiple peptide ratios to derive optimal protein ratios between pairs of samples (Cox *et al.*, 2014), which is widely used as part of the closed source MaxQuant software package. In this note, we present an open-source implementation of the MaxLFQ maximal peptide ratio extraction algorithm for protein quantification in a DIA data processing pipeline called *iq*, short for ion-based protein quantification.

## 2 Implementation

The analysis starts with a long-format data table as exported by Spectronaut version 13.0. The default columns include a primary protein identification column *PG.ProteinGroups*, secondary columns *EG.ModifiedSequence, FG.Charge, F.FrgIon, F.Charge* and quantitative values in *F.PeakArea*. By preparing the input data appropriately, we can use *iq* to accommodate other proteomic data extraction pipelines [see Supplementary Material for examples of processing MaxQuant and OpenSWATH outputs (Röst *et al.*, 2014)].

**Fig. 1.**
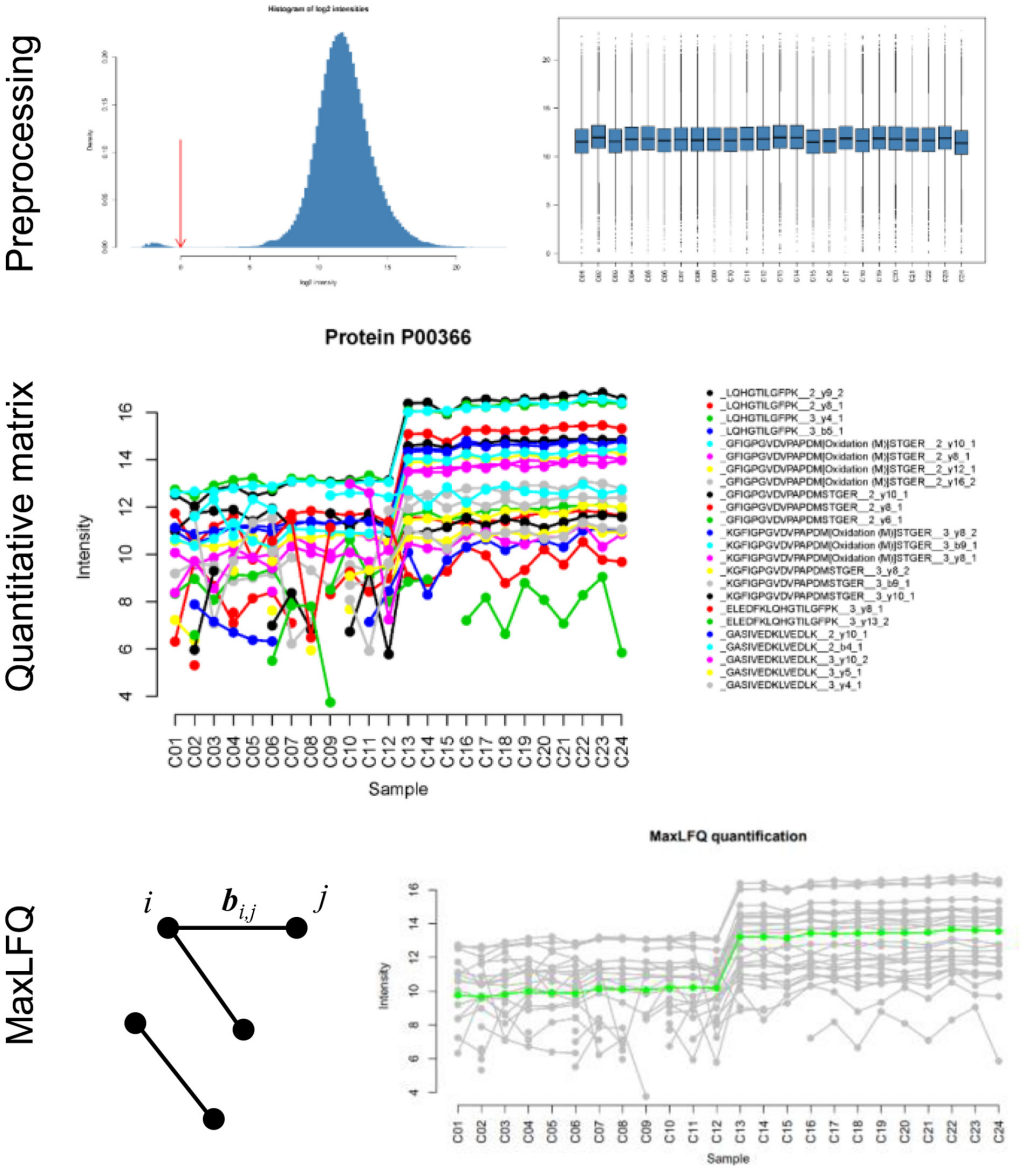
The *iq* pipeline. The preprocessing step performs data filtering and median normalization. Subsequently, a numerical matrix is formed for each protein containing its fragment ion intensities. The MaxLFQ algorithm optimizes ratios $b_{i,j}$ between all pairs of samples *i* and *j*

Figure 1 outlines three main steps in *iq*. First, unique values in the primary column form the list of proteins and a concatenation of the secondary columns determines the fragments used to quantify individual proteins. We provide an option for median normalization of all observed intensities and visualization for quality control.

Second, we enumerate a list of matrices of log2-transformed intensities for all proteins. Let *X* be an observed data matrix for a particular protein with *n* columns for samples and *m* rows for quantified fragment ions. The matrix *X* may have missing values. The aim is to estimate an *n*-vector ***x*** for relative protein abundance across all samples.

The MaxLFQ algorithm obtains ***x*** by minimizing the sum of squared differences between the estimated pairwise sample ratio and the median of the observed ratios

(1) $$\sum_{(i,j)\in \text{valid}\;\text{pairs}}{\left(b_{i,j}-x_{i}+x_{j}\right)}^{2}$$

where $b_{i,j}$ is the median of all pairwise fragment intensity ratios observed between sample *i* and *j* (here differences in log-space). For each fragment, all intensity ratios without missing values are valid pairs.

Third, we solve the above minimization problem as follows. We populate a matrix *A* of size *p *×* n* and a *p*-element vector ***b*** where *p* is the number of valid pairs, and each row of *A* contains all zeroes except for two entries with value of −1 and +1 corresponding to the sample ratio in ***b***. The minimization of (1) becomes an equality constrained least squares

$$\text{min}\;||Ax-b||\;\text{subject}\;\text{to}\;1^{T}x=c$$

where **1** is an *n*-vector of ones and *c* is a scaling constant to preserve the average sum intensity. It can be shown that ***x*** is part of a solution of the system of (n+1) linear equations

(2) $$\left[\begin{array}{cc} 2A^{T}A & 1\\ 1^{T} & 0 \end{array}\right]\left[\begin{array}{c} x\\ z \end{array}\right]=\left[\begin{array}{c} 2A^{T}b\\ c \end{array}\right]$$

where *z* is an auxiliary variable. We employ the R function lsfit to estimate ***x***.

Note that (2) has a unique solution when the square matrix on the left hand side is invertible. A close examination shows that $A^{T}A$ is the Laplacian matrix of the sample graph G whose nodes are the *n* samples, and two nodes are directly connected if there is at least a valid sample pair between them. If G is connected, that is there is a path connecting any two nodes, it can be shown that (2) has a unique solution. This is not the case when G is not connected. Therefore, we implement a recursive spreading algorithm to detect connected components of G. Subsequently, the MaxLFQ algorithm is applied to each connected component. In principle, only samples within a connected component can be compared in downstream analysis due to factors such as different ionization efficiency of different protein fragments. An example is illustrated in Supplementary Figure S7. In short, we have specified the condition under which the algorithm will lead to a valid relative quantification.

Finally, we also implement the topN method, the MeanInt method, and provide a wrapper for the median polish method in *iq*. The package is without any dependency on external R packages.

## 3 Example

We analyze a publicly available dataset used in a benchmark experiment for label-free DDA and DIA proteomics (Bruderer *et al.*, 2015). The dataset for each mode of data acquisition contains 24 runs of 8 biological replicates and 3 technical replicates, in which 12 proteins were spiked in at different concentrations. MaxQuant version 1.6.4.0 is used to process the DDA data, and Spectronaut version 13.0 is used for DIA data. The result of the MaxQuant DDA search is used as a spectral library in the DIA analysis. We use the default Spectronaut long format export with an addition of the columns: *F.ExcludedFromQuantification*, *F.FrgLossType*, *F.FrgIon*, *F.Charge* and *F.PeakArea*. An R script to process the Spectronaut output is as follows:
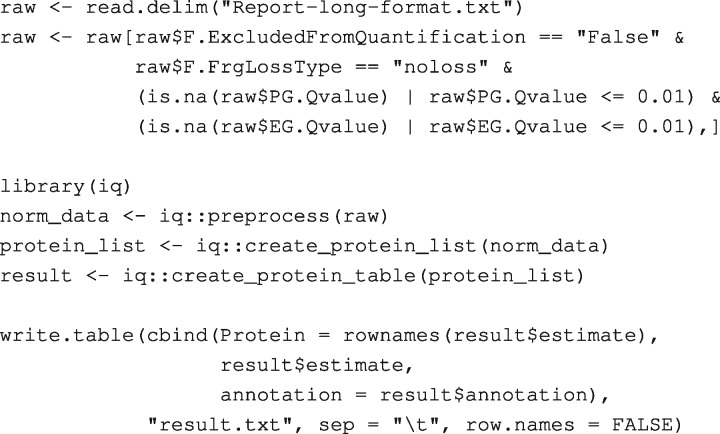


Briefly, the first two statements load the data into R and remove entries not used for quantification. The next four statements load the *iq* package and perform quantification in three steps as described in Section 2. The last statement exports the result to a text file. Protein quantitative visualization used in Figure 1 is created by a function call iq::plot_protein(protein_list$P00366). In the Supplementary Material, we show that the MaxLFQ algorithm compares favorably with other methods in terms of both correlation to the ground-truth values for the 12 spike-in proteins and the stability of background proteins.

## 4 Conclusion

The R package *iq* contains an open-source implementation of the maximal peptide ratio extraction module of MaxLFQ algorithm in a complete pipeline for processing proteomics DIA data. It offers an additional option for protein quantification next to the topN and the median polish approach, while being a direct implementation of a popular algorithm for DDA analysis. We show that optimal relative protein quantification is achieved for comparisons involving the same peptide components.

## Supplementary Material

btz961_Supplementary_Data
