# Supplementary material for: iq: an R package to estimate relative protein abundances from ion quantification in DIA-MS-based proteomics
Source: Bioinformatics. 2020 Jan 7;36(8):2611–3. doi: 10.1093/bioinformatics/btz961 (PMC7178409; doi:10.1093/bioinformatics/btz961)
Supplement: btz961_Supplementary_Data [file btz961_supplementary_data.zip › supplementary.pdf]

Supplementary to "iq: an R package to estimate relative protein abundances from ion quantification in DIA-MS-based proteomics"

Thang V. Pham, Alex A. Henneman, Connie R. Jimenez

1 Example datasets

1.1 The Bruderer dataset using the Spectronaut pipeline

We present an analysis of a publicly available dataset that was used in a benchmark experiment for label-free DDA and DIA proteomics [1]. The raw LC-MS/MS data is available at the public repository PeptideAtlas (http://www.peptideatlas.org/PASS/PASS00589). Each type of acquisition contains 24 runs of 8 biological replicates and 3 technical replicates, with 12 proteins were spiked in at different concentrations.

Three following fasta files are concatenated for the DDA search using MaxQuant version 1.6.4.0 [2]. The sequences of the spike-in proteins are in the file *QS-spike-in-proteins.fasta* on the PeptideAtlas repository. Fasta sequences of the peptides used for retention time alignment (the iRT peptides) are available from Biognosys (https://biognosys.com/). A fasta file of the human proteome release January 2019 is available from Uniprot (http://www.uniprot.org/).

The result of the MaxQuant DDA search is used as a spectral library in Spectronaut version 13.0 to process DIA data. Each sample is assigned to a unique condition in Spectronaut. Subsequently, we use the conditions from C01 to C24 as sample names. We use the default Spectronaut long format export with the addition of columns: *PG.Genes*, *PG.ProteinNames*, *F.ExcludedFromQuantification*, *F.FrgLossType*, *F.FrgIon*, *F.Charge*, and *F.PeakArea*. The first two columns are optional, providing annotations for the quantified proteins in *PG.ProteinAccessions*.

The following script produces a quantified protein table *result.txt* which can be loaded into a spreadsheet application such as Excel.

```r
sp_tab <- read.delim("Spectronaut-report-longformat.txt")

sp_tab <- sp_tab[sp_tab$F.ExcludedFromQuantification == "False" &

                 sp_tab$F.FrgLossType == "noloss" &

                 (is.na(sp_tab$PG.Qvalue) | sp_tab$PG.Qvalue <= 0.01) &

                 (is.na(sp_tab$EG.Qvalue) | sp_tab$EG.Qvalue <= 0.01), ]

sp_norm_data <- iq::preprocess(sp_tab)

sp_protein_list <- iq::create_protein_list(sp_norm_data)

sp_result <- iq::create_protein_table(sp_protein_list)

write.table(cbind(Protein = rownames(sp_result$estimate),

                  sp_result$estimate,

                  annotation = sp_result$annotation),

            "result.txt", sep = "\t", row.names = FALSE)
```

Quality control plots produced in the preprocessing step is shown in **Sup. Fig. 1**. Furthermore, the function *iq::plot_protein()* plots the underlying data for individual proteins. The following code creates the figures in the main text, and **Sup. Fig. 2**.

```r
# Fig. 1 in main text

iq::plot_protein(protein_list$P00366, main = "Protein P00366",

                 cex = 0.6, split = NULL)

iq::plot_protein(rbind(protein_list$P00366,

                       MaxLFQ = iq::maxLFQ(protein_list$P00366)$estimate),

                 main = "MaxLFQ quantification", cex = 0.6,

                 col = c(rep("gray", nrow(protein_list$P00366)), "green"),

                 split = NULL)

# Sup. Fig. 2.

iq::plot_protein(protein_list$P00366, main = "Protein P00366", cex = 0.6)

iq::plot_protein(rbind(protein_list$P00366,

                       MaxLFQ = iq::maxLFQ(protein_list$P00366)$estimate),

                 main = "MaxLFQ quantification", cex = 0.6,

                 col = c(rep("gray", nrow(protein_list$P00366)), "green"))
```

1.2 *The Schubert dataset using the OpenSWATH pipeline*

We downloaded the OpenSWATH-processed data from the publication of Schubert *et al.* [3]. The data is in a long format with fragment ions and their corresponding intensities concatenated in two entries for each peptide. We split these entries into an extended long format, one line for each entry. Subsequently, protein quantification is performed as described in the package vignette. A code snippet is as follows.

```
quant <- as.double(os_tab$aggr_Peak_Area)
short_name <- paste(os_tab$Condition, os_tab$BioReplicate,
                    os_tab$Run, sep = "_")
os_tab <- cbind(os_tab[, c("ProteinName", "FullPeptideName", "Charge",
                           "aggr_Fragment_Annotation")], quant, short_name)
os_norm_data <- iq::preprocess(os_tab,
                               primary_id = "ProteinName",
                               secondary_id = c("FullPeptideName", "Charge",
                                                "aggr_Fragment_Annotation"),
                               sample_id = "short_name",
                               intensity_col = "quant")
os_protein_list <- iq::create_protein_list(os_norm_data)
os_result <- iq::create_protein_table(os_protein_list)
```

2 Comparison of methods on the spike-in dataset

We evaluate the performance of different quantification methods on the set of 12 spike-in proteins where the ground-truth values are available. We calculate the Pearson correlation between the ground-truth and the relative abundance estimated by each method: MaxLFQ, median polish (Tukey, 1977), top3 (using the three most intense fragment ions), top5 (using the five most intense fragment ions), and MeanInt (using all fragment ions). We also include the result of the Spectronaut built-in protein export. **Sup. Fig. 3(A)** shows that all methods perform well. The MaxLFQ algorithm has the highest median correlation value.

Next we calculate the standard deviations of the background proteins for all six methods. **Sup. Fig. 3(B)** shows the boxplots of the standard deviations of proteins quantified in at least half of the samples. Since most of the proteins are stable background, a lower standard deviation is better. Here the MaxLFQ algorithm also performs best.

Note that there are outliers in the result of the top3 method. We can use the protein plotting function to display different quantitative methods for manual examination. For instance, the following creates **Sup. Fig. 4**, showing the quantitative values of a spikein protein using the MaxLFQ method, the top3 method, the Spectronaut built-in method, and the ground truth values. The result of MaxLFQ quantification in green follows the ground truth in gold, while the Spectronaut and top3 results do not, most likely due to an outlier ion _RLDGSLDFK__2_b7_1.

```
# Fig. 4A
iq::plot_protein(protein_list$P12799, main = "Protein P12799", cex = 0.37)
# Fig. 4B
MaxLFQ_estimate <- iq::maxLFQ(sp_protein_list$P12799)$estimate
top3_estimate <- iq::topN(sp_protein_list$P12799)$estimate
top3_estimate <- top3_estimate - mean(top3_estimate) + mean(MaxLFQ_estimate)
sp_protein_report <- read.delim("Spectronaut-protein-report.txt",
                                stringsAsFactors = FALSE, na.strings = "Filtered")
rownames(sp_protein_report) <- sp_protein_report[, "PG.ProteinAccessions"]
sp_quant <- log2(as.numeric(sp_protein_report["P12799", 2:25]))
sp_quant <- sp_quant - mean(sp_quant) + mean(MaxLFQ_estimate)
ground_truth <-  log2(rep(c(200, 125.99, 79.37, 50, 4, 2.52, 1.59, 1), each = 3))
ground_truth <- ground_truth - mean(ground_truth) + mean(MaxLFQ_estimate)
iq::plot_protein(rbind(MaxLFQ = MaxLFQ_estimate,
                       `Spectronaut estimate` = sp_quant,
                       `top3` = top3_estimate,
                       Groundtruth = ground_truth),
```

```
                 main = "P12799 - MaxLFQ, Spectronaut, and top3", cex = 1,
                 col = c("green", "blue", "cyan", "gold"))
```

3 Computing technical and biological correlations for the Schubert dataset

There are 24 samples in the Schubert dataset consisting of biological triplicates of six time points (18 samples) and technical replicates (6 samples). Schubert *et al.* report a median Pearson correlation coefficient of R = 0.95 between biological replicates and R = 0.97 between technical replicates. **Sup. Fig. 5** shows that the correlation of all methods are in the range reported by the paper. For MaxLFQ, the median Pearson correlation coefficient is 0.97 for biological replicates and 0.98 for technical replicates. The top5 method returns the highest reproducibility. Nevetheless, without a ground-truth value for the true reproducibility, we cannot compare the performance. The analysis here demonstrates that the package can support output from the OpenSWATH pipeline [4].

4 Comparison DIA versus DDA

For DDA data, we import the LFQ values from the MaxQuant output, ignoring entries detected in the reversed fasta database. We perform median normalization on intensities in the *evidence.txt* file. Subsequently, we produce a list of proteins as in the case of Spectronaut output in a variable *mq_protein_list* as in the package vignette.

Once the protein list is created, we can perform the MaxLFQ algorithm for DDA as for DIA data. An example for maxLFQ by MaxQuant and *iq* is shown in **Sup. Fig. 6**. An example of the multiple-component situation is illustrated in **Sup. Fig. 7**.

```
# Sup. Fig. 6A
w1 <- iq::maxLFQ(mq_protein_list$A1L0T0)$estimate
w2 <- as.numeric(dda_log2["A1L0T0", ])
w2 <- w2 - mean(w2, na.rm = TRUE) + mean(w1, na.rm = TRUE)
```

```r
tmp <- rbind(mq_protein_list$A1L0T0,
             `MaxLFQ by iq` = w1,
             `MaxLFQ by MaxQuant` = w2)
colnames(tmp) <- sprintf("C%02d", 1:24)
iq::plot_protein(tmp,
                 main = "A1L0T0", cex = 0.8,
                 col = c(rep("gray", nrow(mq_protein_list$A1L0T0)), "green", "blue"))
# Sup. Fig. 6B
w1 <- iq::maxLFQ(mq_protein_list$`O00764-2;O00764;O00764-3`)$estimate
w2 <- as.numeric(dda_log2["O00764-2;O00764;O00764-3", ])
w2 <- w2 - mean(w2, na.rm = TRUE) + mean(w1, na.rm = TRUE)
tmp <- rbind(mq_protein_list$`O00764-2;O00764;O00764-3`,
             `MaxLFQ by iq` = w1,
             `MaxLFQ by MaxQuant` = w2)
colnames(tmp) <- sprintf("C%02d", 1:24)
iq::plot_protein(tmp,
                 main = "O00764-2;O00764;O00764-3", cex = 0.8,
                 col = c(rep("gray", nrow(mq_protein_list$`O00764-2;O00764;O00764-3`)),
                         "green", "blue"))

# Sup. Fig. 7
tmp <- mq_protein_list$`Q13045-2;Q13045-3;Q13045`
colnames(tmp) <- sprintf("C%02d",1:24)
iq::plot_protein(rbind(tmp,
                       MaxLFQ = iq::maxLFQ(tmp)$estimate),
                 main = "O00764-2;O00764;O00764-3",
                 col = c("red", "blue", "cyan", "gold", "yellow", "green"),
                 cex = 0.7)
```

Next we define a measure to assess the difference between DDA and DIA quantification. For each protein, let x and y be two vectors of quantitation for all samples. Since the quantification is relative, we

scale the two vectors to $\overline{x}$ and $\overline{y}$ so that the two means are equal. The difference is defined as average of the absolute differences

$$d(x, y) = \text{mean}(|\overline{x} - \overline{y}|_1)$$

We consider only proteins with solid quantification with more than half of non-missing values (>11 in our example). **Sup. Fig. 8** shows the boxplots of the differences between different quantitative methods for DIA against DDA. It can be seen that the MaxLFQ algorithm outperforms other methods in terms of correspondence to DDA data.

5 References

1. Bruderer, R., Bernhardt, O.M., Gandhi, T., Miladinović, S.M., Cheng, L.-Y., Messner, S., Ehrenberger, T., Zanotelli, V., Butscheid, Y., Escher, C., others: Extending the limits of quantitative proteome profiling with data-independent acquisition and application to acetaminophen-treated three-dimensional liver microtissues. *Molecular & Cellular Proteomics*. 14, 1400–1410 (2015).

2. Cox, J., Mann, M.: MaxQuant enables high peptide identification rates, individualized ppb-range mass accuracies and proteome-wide protein quantification. Nature Biotechnology. 26, 1367 (2008).

3. Schubert OT, K.M., Ludwig C: Absolute proteome composition and dynamics during dormancy and resuscitation of mycobacterium tuberculosis. *Cell Host Microbe*. 18, 96–108 (2015).

4. Röst, H.L., Rosenberger, G., Navarro, P., Gillet, L., Miladinović, S.M., Schubert, O.T., Wolski, W., Collins, B.C., Malmström, J., Malmström, L., others: OpenSWATH enables automated, targeted analysis of data-independent acquisition MS data. *Nature Biotechnology*. 32, 219 (2014).
