# Supplementary material for: iq: an R package to estimate relative protein abundances from ion quantification in DIA-MS-based proteomics
Source: Bioinformatics. 2020 Jan 7;36(8):2611–3. doi: 10.1093/bioinformatics/btz961 (PMC7178409; doi:10.1093/bioinformatics/btz961)
Supplement: btz961_Supplementary_Data [file btz961_supplementary_data.zip › supplementary-figures.pdf]

(A)

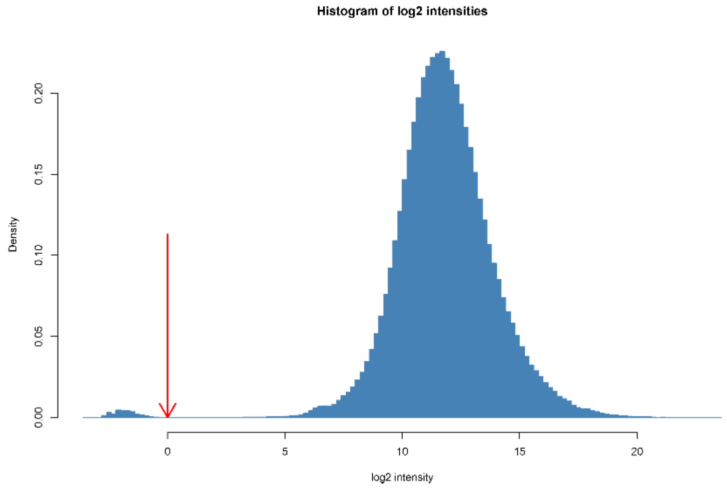

(B)

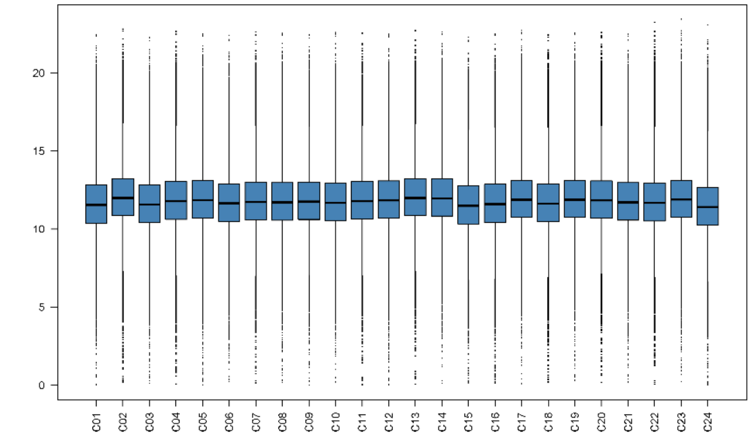

(C)

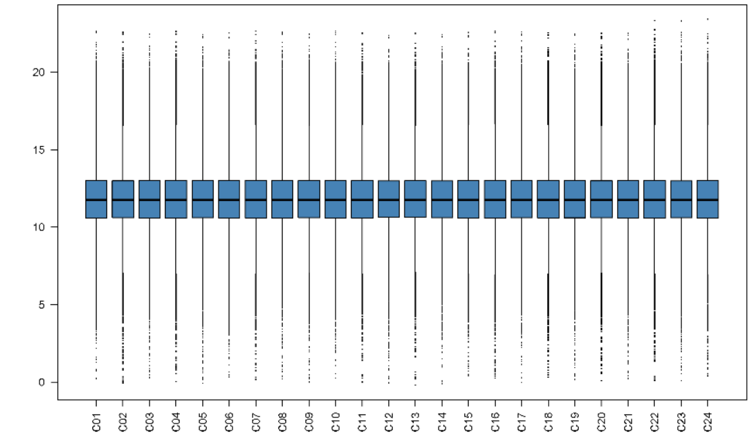

Sup. Fig. 1: (A) Distribution of log2 intensities. A cutoff value of zero is applied (red arrow). (B) Boxplots of intensities per sample. (C) Boxplots of intensities after median normalization.

(A)

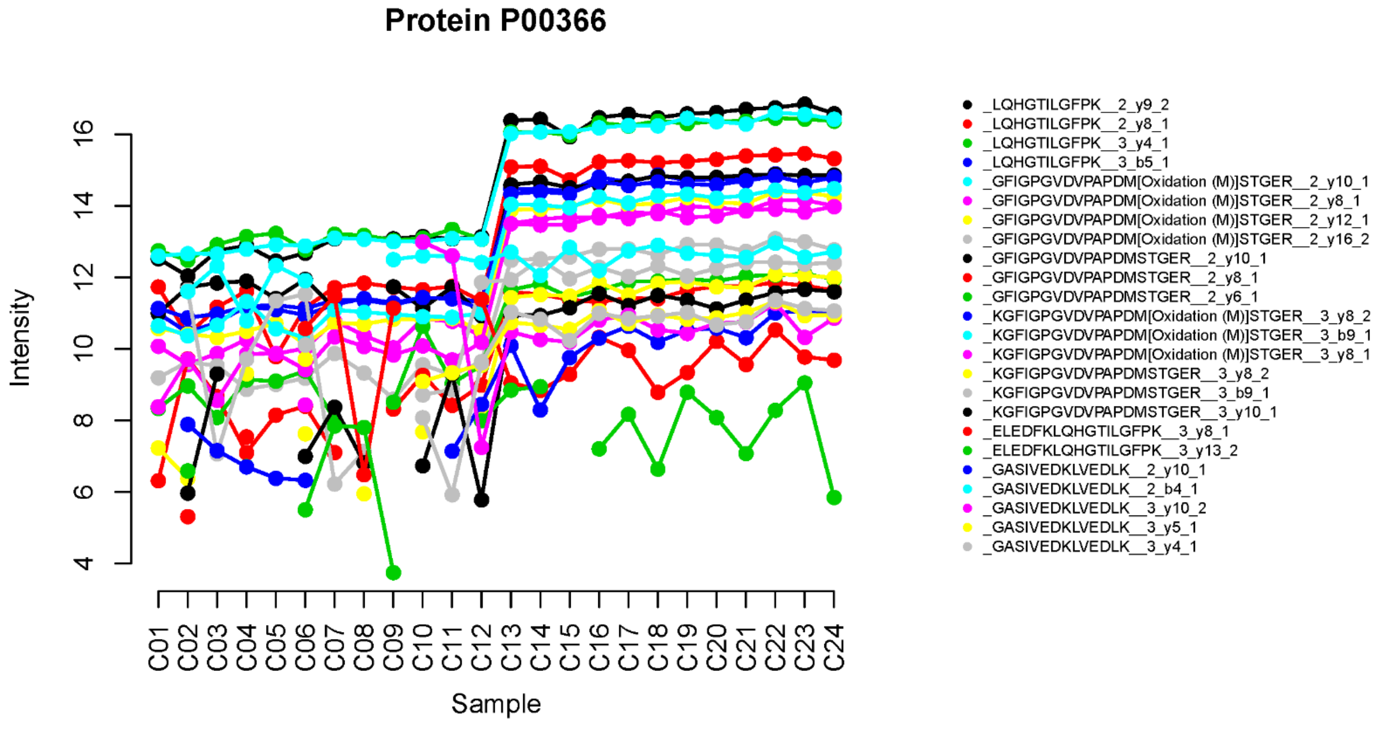

(B)

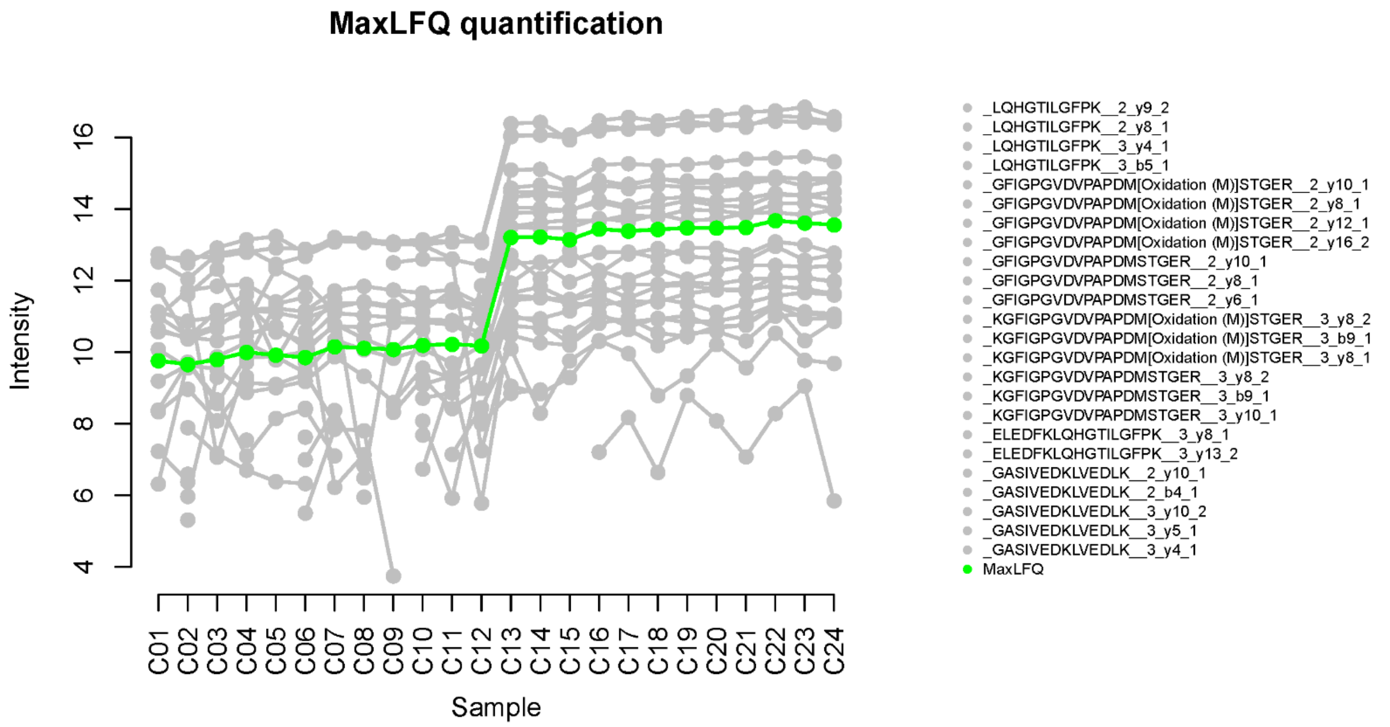

Sup. Fig. 2: Visualization using the *iq* protein plotting function. (A) underlying data for the protein P00366. (B) Result of MaxLFQ quantification in green.

(A)

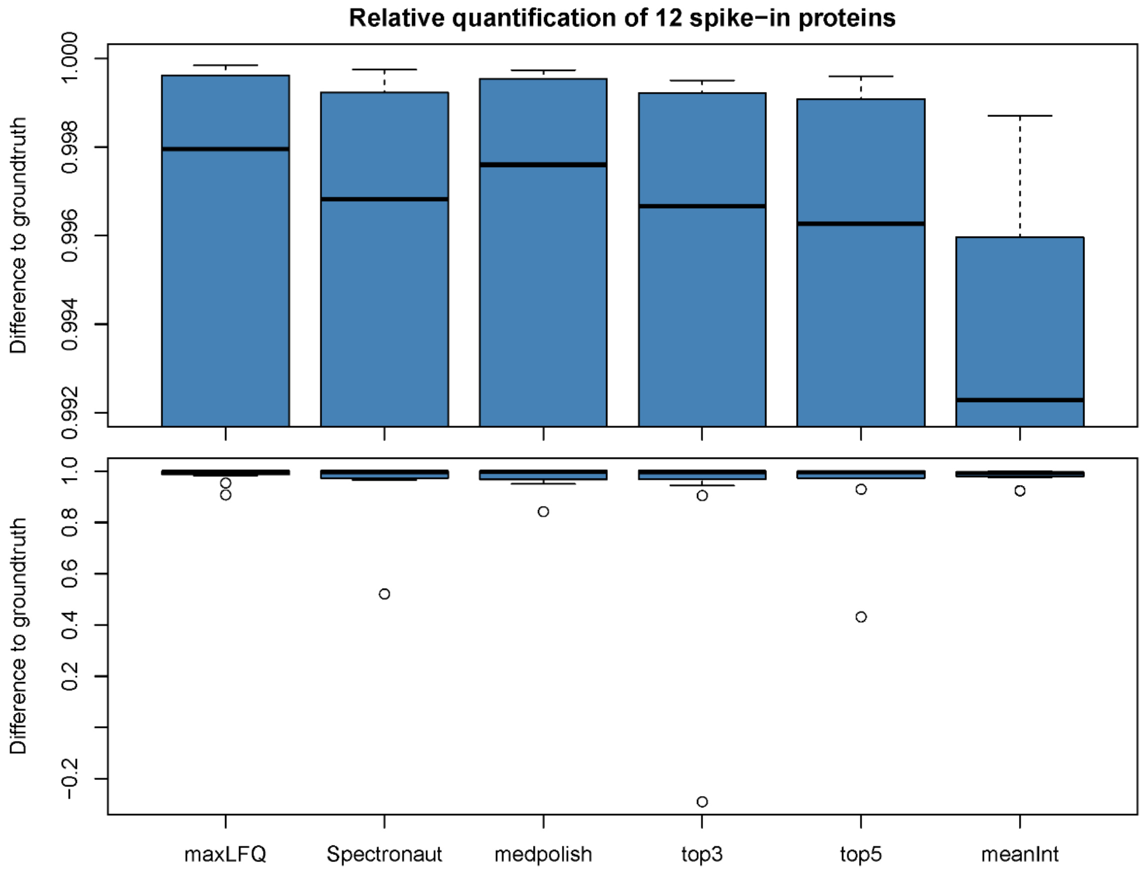

(B)

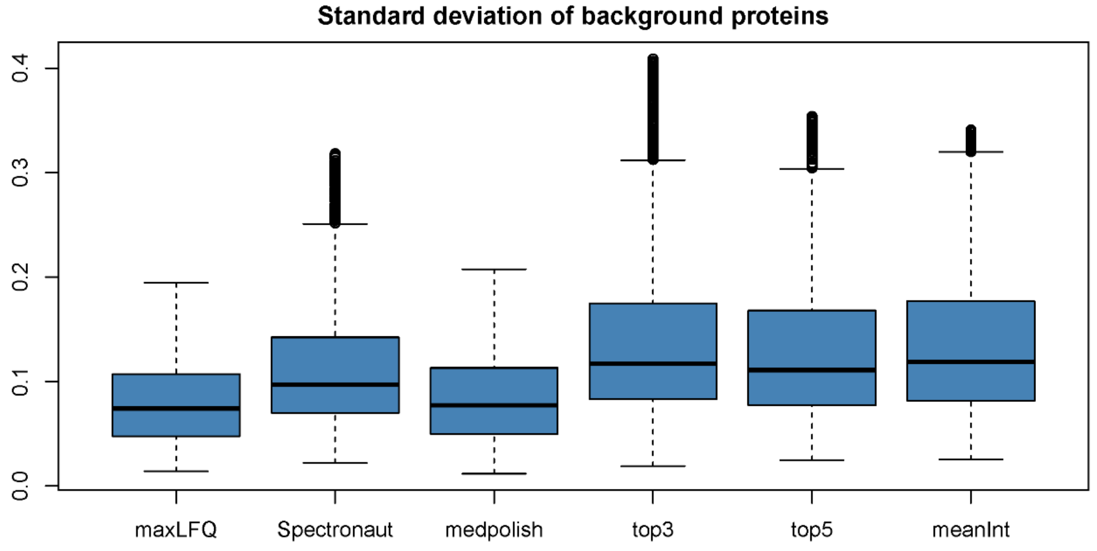

Sup. Fig. 3: (A) (bottom) Boxplots of Pearson correlations of the results of six quantitation methods to the ground-truth values for 12 spike-in proteins. MaxLFQ is implemented by *iq*. Spectronaut is the result returned by Spectronaut built-in protein quantification. Other methods are medpolish (median polish), top3 (using the three most intense fragment ions), top5 (using the five most intense fragment ions), and meanInt (using all fragment ions). (top) zooming in on the median values. (B) Boxplots of the standard deviations of the top 90% of the most stable proteins for six different methods on the DIA dataset. The MaxLFQ algorithm (median = 0.074) is slightly better than the median polish method (median = 0.077).

(A)

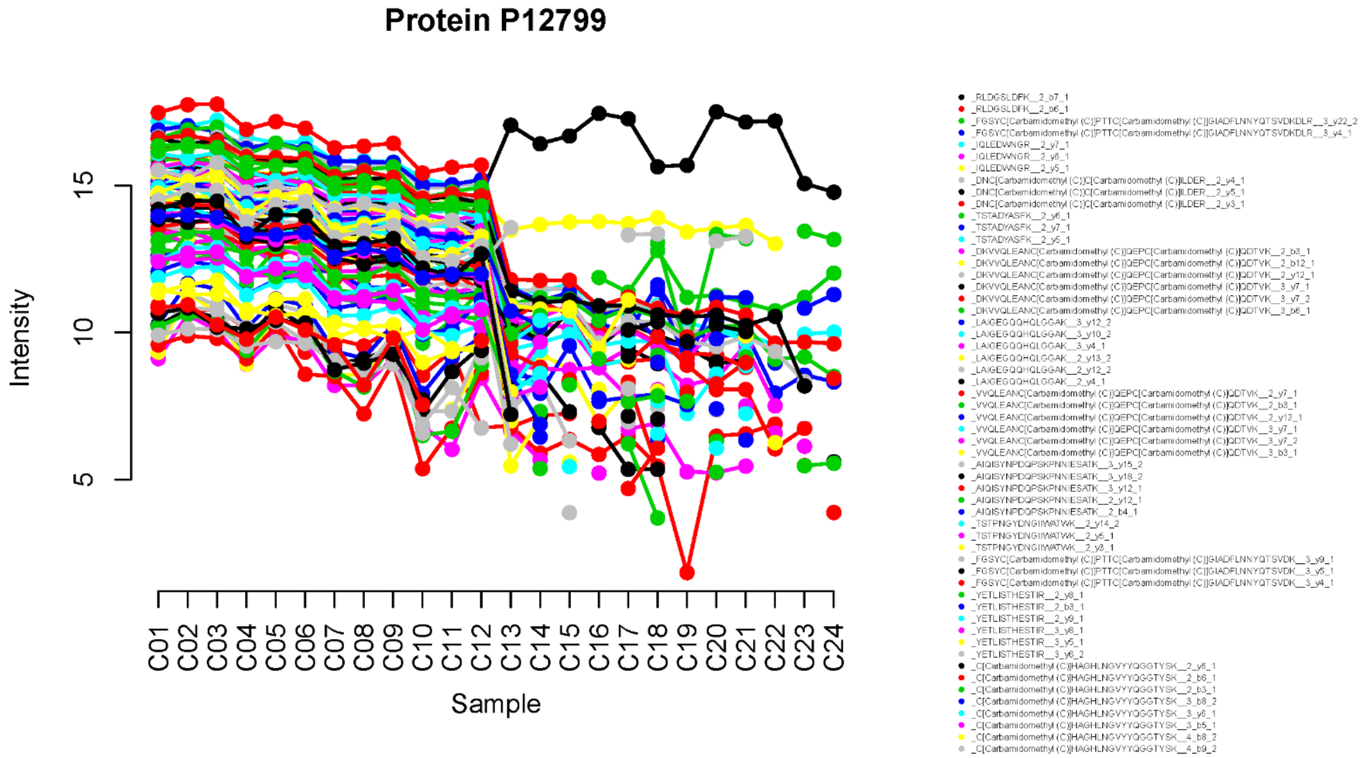

(B)

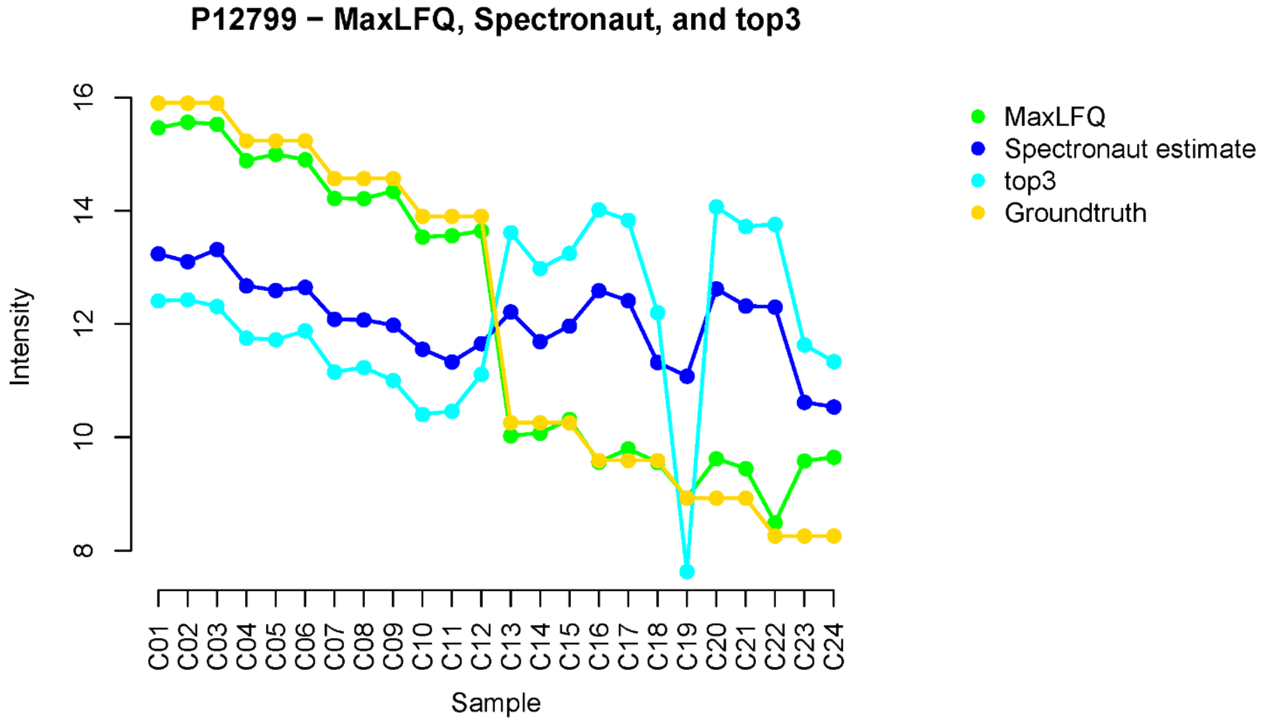

Sup. Fig. 4: (A) underlying data for a spike-in protein P12799. (B) The result of MaxLFQ quantification in green follows the ground truth in gold, while the built-in Spectronaut estimate and the top3 method do not, most likely due to an outlier ion _RLDGSLDFK__2_b7_1.

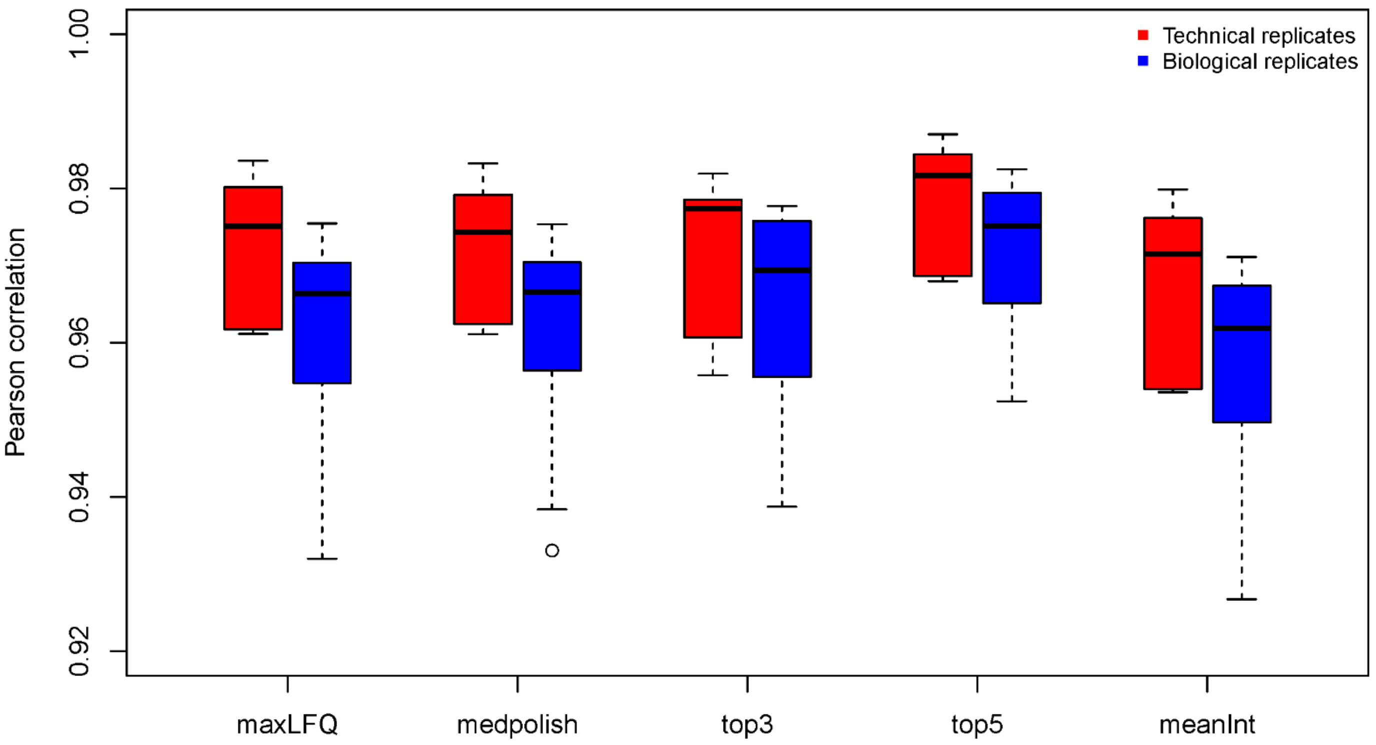

Sup. Fig. 5: Boxplots of Pearson correlation between biological and technical replicates of the OpenSWATH-processed Schubert dataset (see text).

(A)

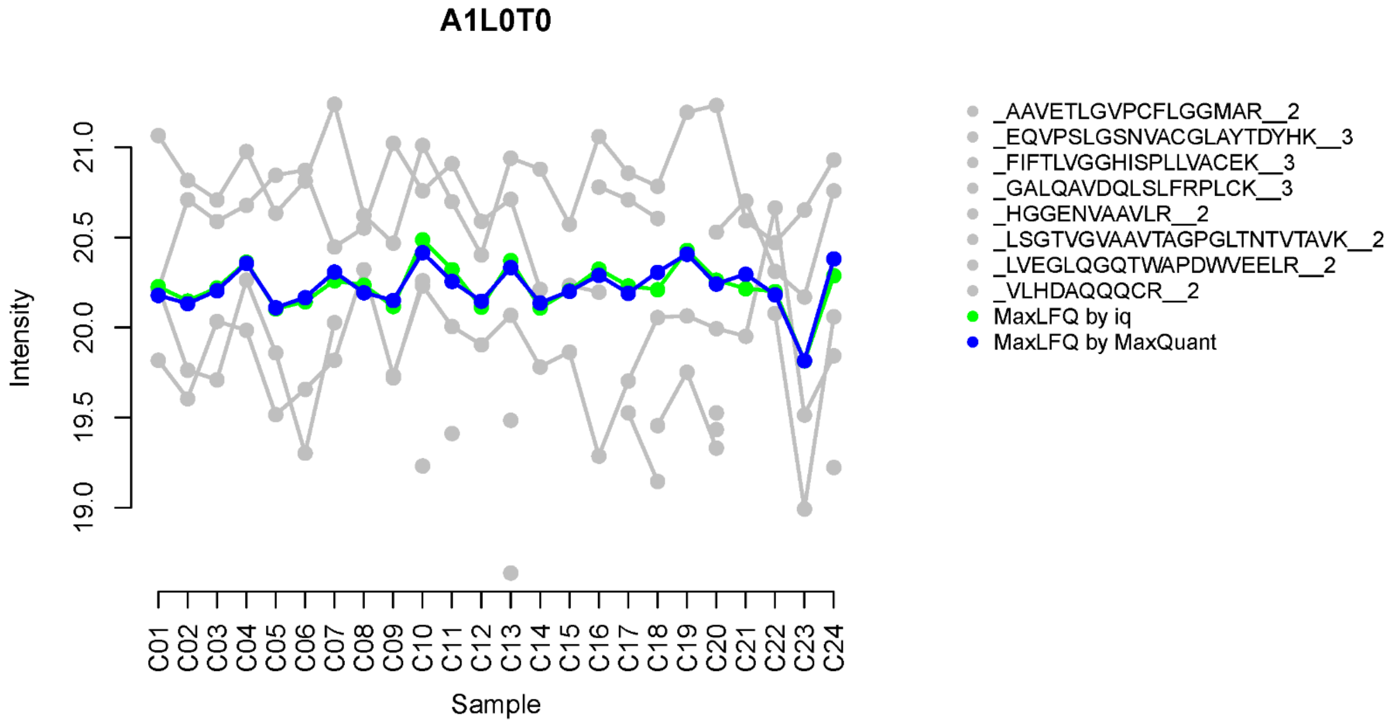

(B)

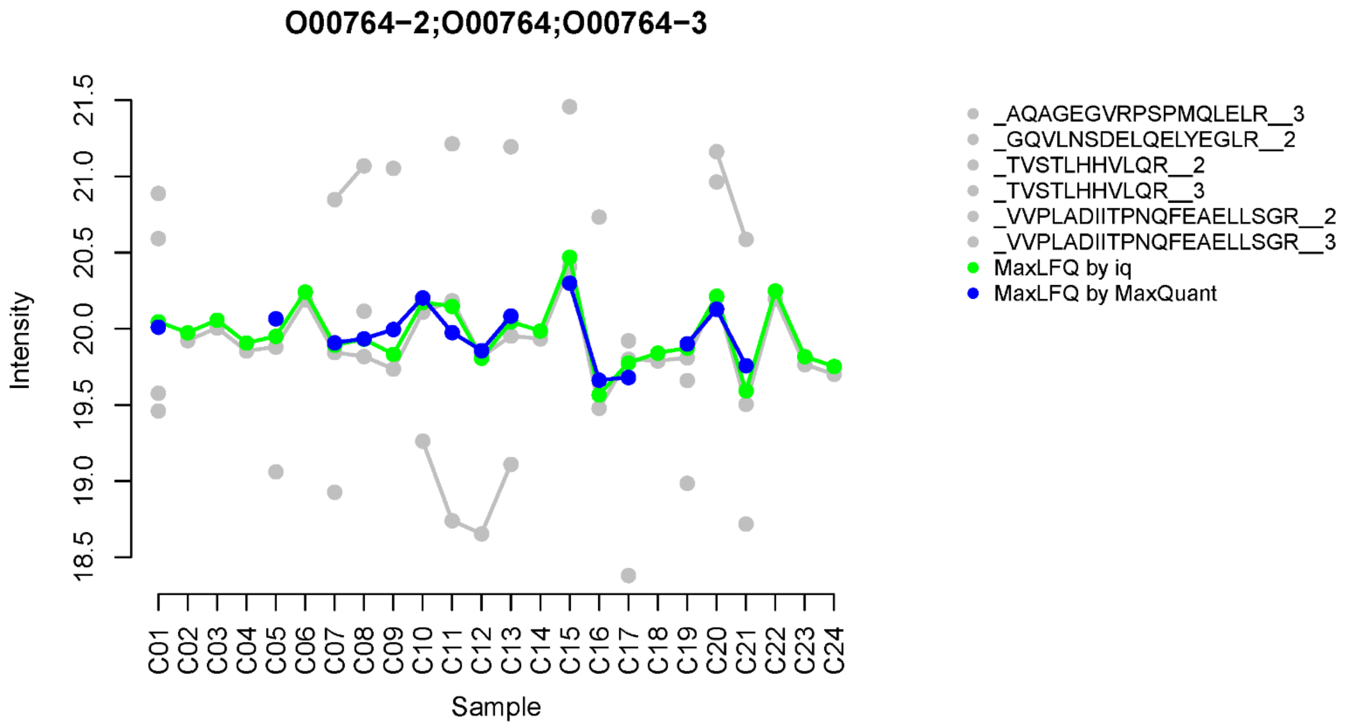

Sup. Fig. 6: (A) An example of MaxLFQ quantification using MaxQuant and *iq* on the DDA dataset. There is a small difference between the two implementations. (B) There are more missing values in MaxQuant implementation. This might be due to the threshold on the number of ratios for a quantification in MaxQuant.

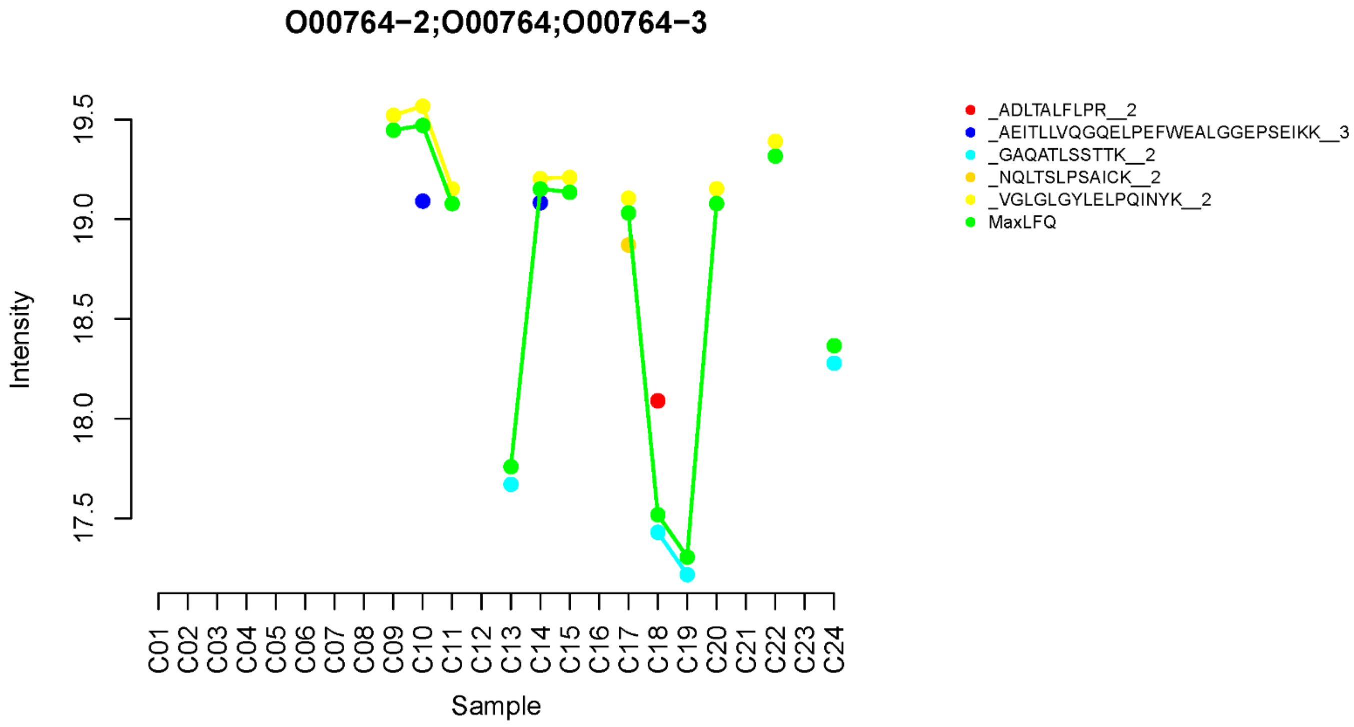

Sup. Fig. 7: An example of the MaxLFQ algorithm on the DDA dataset.
Samples C09, C10, C11, C14, C15, C17, C20 and C22 are in a connected component, whereas samples C13, C18, C19 and C24 form another connected component.

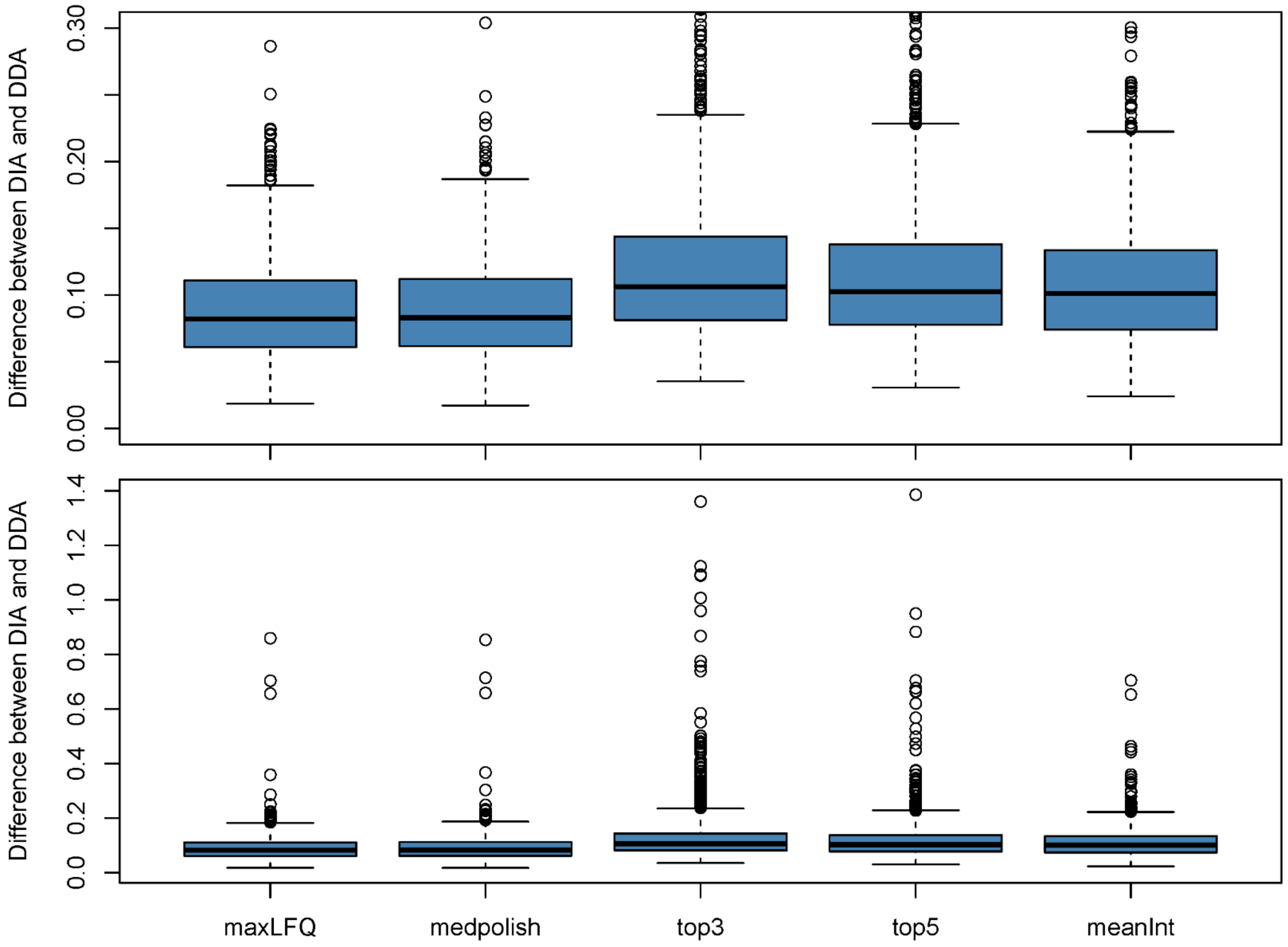

Sup. Fig. 8: Boxplots of differences between DIA and DDA quantification (the top figure zooms in on the median values). For each protein, the average of the absolute differences between values measured in DIA and DDA is calculated (see Supplementary text for detail). A lower value indicates a higher similarity. The MaxLFQ and median polish methods perform equally well. The median difference of MaxLFQ is slightly better (0.082 vs 0.083). Both are better than top3, top5, and MeanInt.
